# From the bench to the bedside: Spinal cord regeneration, niacin for stroke, magnetic nanoparticles, stimulation for epilepsy, role of galanins in epilepsy, functions of the supramarginal gyri, and the role of inflammation in postoperative cognitive disturbances

**DOI:** 10.4103/2152-7806.71985

**Published:** 2010-10-20

**Authors:** Jason S. Hauptman, Michael Safaee

**Affiliations:** Intellectual and Developmental Disabilities Center and Department of Neurosurgery, Geffen School of Medicine at UCLA, Los Angeles, CA, USA

## UNLOCKING THE POTENTIAL FOR CORTICOSPINAL NEURONS TO REGENERATE FOLLOWING INJURY

Regeneration of the corticospinal tract following spinal cord injury[1] could provide tremendous recovery potential to patients who have lost the ability for volitional movement. At present, however, studies have shown that adult corticospinal neurons are relatively resistant to techniques promoting regrowth. In this study, the authors investigate the role of *Pten*, a key regulatory enzyme that is responsible for controlling the activity of *mTOR* (a complex of proteins responsible for cellular growth and proliferation). The researchers found that the reduced ability of corticospinal neurons to sprout new projections following injury coincided with a reduction in *mTOR* activity. Furthermore, when *mTOR* levels were increased by deleting *Pten*, axon sprouting from corticospinal neurons substantially increased after spinal cord crush lesions. These new axons also formed synapses, though the functionality of these synapses and their postsynaptic contacts remain to be seen. In all, this work suggests that mature corticospinal neurons can be induced to form robust new axonal projections via *Pten* deletion, following injury. In the future, studies will need to be conducted to see if this translates into faster recovery of motor function *in vivo*. Future work into discovering agents that can pharmacologically manipulate the function of *Pten* may play a critical role in recovery following corticospinal injury.

## NIACIN THERAPY MAY ENHANCE NEUROLOGICAL RECOVERY FOLLOWING STROKE

Niacin, a medication commonly used clinically to increase high-density lipoprotein cholesterol (HDL-C), may also have a role in improving functional outcome following stroke.[2] Because HDL-C is critical for maintaining cell membrane integrity and also has effects on synaptic function and plasticity, researchers proposed that it might also increase neuronal recovery and synapse formation in animals that experience stroke. In this study, rats underwent middle cerebral artery (MCA) occlusion for 2 hours. A cohort was given a delayed-release form of niacin (called Niaspan) starting 24 hours after stroke for a total of 2 weeks. Using a variety of histology techniques, the authors showed that the brain tissue adjacent to the ischemic core had greater markers for axons and synapses and fewer proteins that inhibit axonal growth in the group treated with Niaspan. Furthermore, they demonstrated increased brain-derived neurotrophic factor (BDNF) and its receptor TrkB in the Niaspan-treated cohort. BDNF has been suggested to assist in neuronal survival and regeneration following injury. Interestingly, when the authors applied either niacin or HDL to cultured neurons that underwent oxygen-glucose deprivation (OGD), they also found the increase in BDNF and TrkB. This suggests that the changes in BDNF/TrkB may be caused by the increase in HDL due to niacin treatment. In this same *in vitro* model, they showed that niacin and HDL both increase neurite outgrowth, an effect that was reversed by application of a TrkB inhibitor. Consequently, this work points to the fact that niacin-induced increases in HDL are linked to increases in BDNF/TRkB-mediated neurite outgrowth. One question that remains, however, is whether this neurite outgrowth and synaptogenesis leads to functional synaptic contacts and restored neural circuitry. This seems like a relatively easy study to translate to the bedside, since niacin has low-morbidity and beneficial effects on stroke risk factors. It would be interesting to see if niacin treatment translates into better neurological outcomes, strengthening the case that this neurite outgrowth translates into functional recovery.

## USING MAGNETIC NANOPARTICLES TO TREAT BRAIN TUMORS

The advent of nanoparticles carrying pharmacological therapeutics has been an exciting development in contemporary neuroscience. Getting these nanoparticles across the blood-brain barrier (BBB) has been challenging, though recently focused ultrasound (FUS) has been found to be able to locally disrupt the BBB to allow different agents through. Another challenge has been how to evaluate how much agent enters the central nervous system (CNS), a problem that has been addressed using magnetic nanoparticles that can be visualized using magnetic resonance imaging (MRI). Another advantage of these magnetic nanoparticles is the ability to perform magnetic targeting (MT), whereby magnet application can guide the nanoparticles to particular regions. In this study, the authors used focused ultrasound to deliver magnetic nanoparticles to the CNS while monitoring their delivery with MRI. First, they characterized these magnetic nanoparticles and conjugated them to epirubicin, a cytotoxic drug used to treat cancer. When these nanoparticles were added to cell cultures, the cells took up the nanoparticles via endocytosis and underwent apoptosis. Furthermore, using MT, cell death was concentrated at areas nearest to the magnet. In *in vivo* rat studies, the authors demonstrated that FUS and MT resulted in magnetic nanoparticle deposition in brain regions nearest to the magnet, as demonstrated by MRI. Finally, the authors created animals bearing brain tumors[3] and showed that by applying MT for 6 hours after FUS treatment, a significant amount of epirubicin was deposited in the tumor-bearing region. This resulted in significant reductions in tumor progression. Overall, this novel therapy appears to be very promising for targeted delivery of drugs to specific regions of the brain. That being said, it will be limited by the fundamental pathology of some primary brain tumors: the infiltrative process of gliomas. Also, as this technology continues to evolve, it is easy to imagine a variety of applications in neurosurgical oncology, epilepsy neurosurgery, and functional neurosurgery.

## SUPPRESSING SEIZURE ACTIVITY WITH *IN VIVO* LOW FREQUENCY ELECTRICAL STIMULATION

Deep brain stimulation has come into focus as a potential therapeutic modality in patients with intractable epilepsy. For instance, in March 2010, a large clinical study examining the efficacy of anterior thalamic nucleus high frequency stimulation in the treatment of refractory epilepsy was published.[5] One potential advantage to the low frequency stimulation (LFS) alternative, however, is less current injection that requires less power usage and less potential tissue disruption. In this study,[4] the authors performed low frequency stimulation of the bilateral ventral hippocampal commissure (VHC) in mice with an ion channel mutation that predisposes them to epilepsy. Following LFS, the animals experienced a ~20% decrease in mean seizure frequency and a ~35% reduction in seizure duration. Interestingly, these numbers are similar to the clinical study dealing with anterior thalamic stimulation, which demonstrated ~30% reduction in seizure frequency during blinded stimulation.[5] In the human study, there were significant chronic effects of stimulation as well, with a ~56% reduction in median seizure frequency in the 2 years following implantation. LFS of the VHC deserves further consideration as a therapeutic modality for intractable epilepsy, though more long-term data would be useful to see if the antiepileptic benefits of stimulation continued beyond the 12 days studied. Also, it is not clear if LFS of the VHC could result in untoward neuropsychological side effects. In one animal, stimulation actually resulted in an increase in seizure frequency. One must wonder if this was due to electrode positioning and stimulation of adjacent hippocampal structures.

## GALANIN RECEPTORS: A NOVEL TARGET FOR ANTIEPILEPTIC DRUGS

Galanin, a neuropeptide found to be involved in multiple physiological processes including hippocampal epilepsy, has been proposed as a novel target for anticonvulsant therapy.[6] Previous seizure models have demonstrated that enhancement of galanin neurotransmission results in less seizures and delayed development of epilepsy, while animals that lack galanin are afflicted with more severe epilepsy than controls. Since the effects of galanin appear to be mediated through the receptors GalR1 and GalR2, the authors describe a novel compound that acts as a positive allosteric modulator (enhances receptor function) of GalR2. This agent, CYM2503, potentiated galanin’s effects on the GalR2 receptor without affecting GalR1 signaling. Using the pilocarpine model of acute epilepsy, the authors found that CYM2503 pretreatment significantly delayed the onset of seizures approximately fivefold. This was substantiated by a second model of generalized tonic–clonic seizures, which resulted in a similar delay in seizure induction. In a final model of status epilepticus, CYM2503 increased latency to status epilepticus as well as conferred an extremely significant survival benefit. This is a particularly exciting study and opens the door to future work characterizing the role of galanin receptors in epilepsy and developing novel antiepileptic approaches.

## DAMAGE TO THE LEFT INFERIOR PARIETAL LOBE ASSOCIATED WITH CONDUCTION APHASIA

Conduction aphasia, defined as the inability to repeat speech, is common in patients with left hemisphere stroke and resultant aphasia. While this deficit is classically characterized by injury to the left arcuate fasciculus, recent evidence has suggested that it may actually be caused solely by injury to the left inferior parietal cortex.[7] In this study, patients who had acute left hemisphere stroke were examined using a combination of MRI and aphasia batteries. Specific MRI protocols included diffusion-weighted and perfusion-weighted sequences. This type of analysis is considered “lesion-behavior mapping.” The authors found that conduction aphasia correlated strongly to damage to the inferior aspect of the left supramarginal gyrus as well as the temporal-parietal junction. This study adds to our knowledge regarding the role of the parietal cortex in speech production as well as highlights the use of lesion-behavior mapping to determine the functions of different brain regions. It also may assist the neurosurgeon in prognosticating neuropsychological risks and outcomes following approaches to parietal lesions (and more specifically lesions involving the supramarginal gyrus).

## BILATERAL SUPRAMARGINAL GYRI FOUND TO BE CRUCIAL FOR PHONOLOGICAL PROCESSES

In addition to the lesion-behavior mapping study (see above) pinpointing conduction aphasia to the inferior portion of the left supramarginal gyrus, work has also suggested that the supramarginal gyri (SMG) are critical for processing the phonology (sounds) of words (as opposed to their semantics, or meaning).[8] In this study, the authors used a combination of transcranial magnetic stimulation (TMS) to disrupt unilateral SMG and phonological word tasks in order to determine the functional significance of the contralateral SMG. The authors found that regardless of which side the TMS was performed on, reaction times to phonological tasks increased, while reaction times to semantic tasks did not. This effect was specific for SMG inactivation, since inactivation of the angular gyri did not result in the same outcome. As an interesting side note, TMS of the angular gyri did not disrupt semantic processing, a surprising finding considering the existing data which suggest that semantic word tasks rely on the angular gyri. Since no effects were noticed with regard to which side’s SMG was inactivated with TMS, the authors then constructed intensity–effect curves for each side to see if the same intensity of TMS would result in the same magnitude of effect on phonological word tasks. They found that while increasing the TMS intensity resulted in increases in reaction times, the effects were comparable between the left and right SMGs. This study not only demonstrates the utility of TMS in delineating the functionality of different brain regions but also shows the bilaterality of function in the SMG. Lesions of the SMG may result in subtle neuropsychological effects regardless of hemisphere.

## INFLAMMATION FOLLOWING SURGERY MAY LEAD TO POSTOPERATIVE COGNITIVE DISTURBANCES

Postoperative cognitive dysfunction (POCD), characterized by impairments in memory, attention, consciousness, and sleep cycle, is most common in elderly patients after surgery involving either regional or general anesthesia.[9] In this study, Cibelli *et al*. investigated the role of the cytokine interleukin-1β in hippocampal inflammation and cognitive deficits. Mice underwent either an orthopedic procedure under general anesthesia or anesthesia alone. In those that underwent surgery, some were given either minocycline (an antimicrobial with anti-inflammatory properties), enrofloxacin (an antimicrobial similar to minocycline but without anti-inflammatory properties), or an IL-1 receptor antagonist. The authors found that surgery significantly increased serum and hippocampal IL-1β levels, peaking ~6 hours postoperatively. Minocycline administration blunted this cytokine increase in both serum and hippocampal tissue, while enrofloxacin did not. Furthermore, surgery induced microgliosis in the hippocampus, an effect also prevented by the administration of minocycline only. In behavior studies examining hippocampal function, mice that underwent surgery displayed hippocampal learning deficits that were also alleviated by the administration of minocycline. In this case, though, one has to wonder if minocycline may confound the testing due to anti-inflammatory medications’ effects on pain perception. In the final set of experiments, the authors performed surgery on IL-1 receptor null animals or animals treated with the IL-1 receptor antagonist. In these animals, no postoperative surge in cytokine release was detected, no microgliosis in the hippocampus occurred, and no hippocampal-learning deficits were noted on behavioral testing. This interesting study indicated that inflammation plays a crucial role in POCD, and suggests that pretreatment with an anti-inflammatory agent may prevent its occurrence.

## References

[1] Liu K, Lu Y, Lee JK, Samara R, Willenberg R, Sears-Kraxberger I (2010). PTEN deletion enhances the regenerative ability of adult corticospinal neurons. Nat Neurosci.

[2] Cui X, Chopp M, Zacharek A, Roberts C, Buller B, Ion M (2010). Niacin treatment of stroke increases synaptic plasticity and axon growth in rats. Stroke.

[3] Liu HL, Hua MY, Yang HW, Huang CY, Chu PC, Wu JS (2010). Magnetic resonance monitoring of focused ultrasound/magnetic nanoparticle targeting delivery of therapeutic agents to the brain. Proc Natl Acad Sci U S A.

[4] Kile KB, Tian N, Durand DM (2010). FULL-LENGTH ORIGINAL RESEARCH: Low frequency stimulation decreases seizure activity in a mutation model of epilepsy. Epilepsia.

[5] Fisher R, Salanova V, Witt T, Worth R, Henry T, Gross R (2010). Electrical stimulation of the anterior nucleus of thalamus for treatment of refractory epilepsy. Epilepsia.

[6] Lu X, Roberts E, Xia F, Sanchez-Alavez M, Liu T, Baldwin R (2010). GalR2-positive allosteric modulator exhibits anticonvulsant effects in animal models. Proc Natl Acad Sci U S A.

[7] Fridriksson J, Kjartansson O, Morgan PS, Hjaltason H, Magnusdottir S, Bonilha L (2010). Impaired speech repetition and left parietal lobe damage. J Neurosci.

[8] Hartwigsen G, Baumgaertner A, Price CJ, Koehnke M, Ulmer S, Siebner HR Phonological decisions require both the left and right supramarginal gyri. Proc Natl Acad Sci U S A.

[9] Cibelli M, Fidalgo AR, Terrando N, Ma D, Monaco C, Feldmann M (2010). Role of interleukin-1beta in postoperative cognitive dysfunction. Ann Neurol.

